# P-1366. Pulmonary Disease Caused by Non-Tuberculous Mycobacteria in Niger Republic

**DOI:** 10.1093/ofid/ofaf695.1553

**Published:** 2026-01-11

**Authors:** Abdourahamane Yacouba, Bassirou Souleymane, Alphazazi Soumana, Zelika Hamidou Harouna, Saidou Mamadou

**Affiliations:** Unversité Abdou Moumouni / Department of Applied biologicals sciences, Niamey, Niger, Niger, Niamey, Niger; Damien Foundation, Niamey, Niamey, Niger; Programme National de Lutte contre la Tuberculose, Ministère de la Santé Publique, Niamey, Niamey, Niger; Laboratoire National de Reference sur la tuberculose, Niamey, Niamey, Niger; Unversité Abdou Moumouni / Department of Applied biologicals sciences, Niamey, Niger, Niger, Niamey, Niger

## Abstract

**Background:**

Disease caused by non-tuberculous mycobacteria (NTMs) shares many clinical features with tuberculosis (TB), challenging differential diagnosis. GeneXpert currently used for TB screening only detects *Mycobacterium tuberculosis* complex. This study aimed to explore the causes of microscopy-positive but Xpert-negative results in patients with chronic lung disease in Niger.

**Methods:**

This descriptive cross-sectional study was conducted between July 2022 and June 2023 at the TB National Reference Laboratory in Niger, in collaboration with the Institute of Tropical Medicine, Belgium. It included any patient previously treated for TB presenting with a microscopy-positive but Xpert-negative result, for whom sputum samples were cultured on solid medium. A duplex quantitative real-time polymerase chain reaction (qPCR) was performed, targeting the *IS16110* gene to detect the MTBc and the *16S rRNA* gene to detect all species of the *Mycobacterium* genus. Twelve species were tested for Drug susceptibility testing according to the CLSI 2023 standard.

**Results:**

We included 59 patients who were predominantly male (sex ratio = 5.5), with an average age of 49.3 (± 14.8) years, and a mean BMI of 17.4 (± 3.15) kg/m^2^. Respiratory signs were found in 94.9%, whereby productive cough (71.4%) and dyspnea (59%) predominated. Majority of patients (93%) had abnormal chest X-rays, of which 50 had bilateral and 5 unilateral cavitary lesions. Twenty-one different NTMs species were identified, majority belonging to the *M. avium* complex, followed by *M. palustre*. All NTM species tested for DST were susceptible to clarithromycin (12/12) and amikacin (12/12).

**Conclusion:**

NTMs are increasingly isolated from previously treated TB patients in Niger. Isolation and rapid identification of these mycobacteria and assessing their clinical relevance are important, as the treatment strategies for tuberculosis and respiratory NTM infections differ.

**Disclosures:**

All Authors: No reported disclosures

